# Production, active staining and gas chromatography assay analysis of recombinant aminopeptidase P from *Lactococcus lactis* ssp. *lactis* DSM 20481

**DOI:** 10.1186/2191-0855-2-39

**Published:** 2012-08-01

**Authors:** Timo Stressler, Thomas Eisele, Michael Schlayer, Lutz Fischer

**Affiliations:** 1 Institute of Food Science and Biotechnology, Department of Biotechnology, University of Hohenheim, Garbenstr. 25, D-70599, Stuttgart, Germany

**Keywords:** *Lactococcus lactis*, Aminopeptidase P, PepP, Gas chromatographic assay, Activity staining, LPP

## Abstract

The aminopeptidase P (PepP, EC 3.4.11.9) gene from *Lactococcus lactis* ssp*. lactis* DSM 20481 was cloned, sequenced and expressed recombinantly in *E. coli* BL21 (DE3) for the first time. PepP is involved in the hydrolysis of proline-rich proteins and, thus, is important for the debittering of protein hydrolysates. For accurate determination of PepP activity, a novel gas chromatographic assay was established. The release of L-leucine during the hydrolysis of L-leucine-L-proline-L-proline (LPP) was examined for determination of PepP activity. Sufficient recombinant PepP production was achieved via bioreactor cultivation at 16 °C, resulting in PepP activity of 90 μkat_LPP_ L_culture_^-1^. After automated chromatographic purification by His-tag affinity chromatography followed by desalting, PepP activity of 73.8 μkat_LPP_ L_culture_^-1^ was achieved. This was approximately 700-fold higher compared to the purified native PepP produced by *Lactococcus lactis* ssp*. lactis* NCDO 763 as described in literature. The molecular weight of PepP was estimated to be ~ 40 kDa via native-PAGE together with a newly developed activity staining method and by SDS-PAGE. Furthermore, the kinetic parameters *K*_*m*_ and *V*_*max*_ were determined for PepP using three different tripeptide substrates. The purified enzyme showed a pH optimum between 7.0 and 7.5, was most active between 50°C and 60°C and exhibited reasonable stability at 0°C, 20°C and 37°C over 15 days. PepP activity could be increased 6-fold using 8.92 mM MnCl_2_ and was inhibited by 1,10-phenanthroline and EDTA.

## Introduction

Aminopeptidase P (PepP; EC 3.4.11.9) is an exopeptidase that removes the N-terminal amino acid at the P_1_ position [nomenclature following [Schechter and Berger (1967])] from a peptide when proline is in the P_1_´ position ([Cunningham and O'Connor 1997]). Several enzymes with the specificity of PepP have been found in species such as *Escherichia coli* ([Yaron and Mlynar 1968]; [Yoshimoto et al. 1988b]*)Neisseria gonorrhoeae* ([Chen and Buchanan 1980]), *Thermococcus* sp. strain NA1 ([Lee et al. 2006]), *Lactococcus lactis* ssp. *lactis* NCDO 763 ([Mars and Monnet 1995]), *Lactococcus lactis* ssp. *cremoris* AM2 ([McDonnell et al. 1997]) and tissues from several mammalian species ([Hooper et al. 1990]; [Harbeck and Mentlein 1991]). While the physiological role of PepP in bacteria is unclear, mammalian PepP is involved in processes such as the regulation of biologically active peptides, including substance P and bradykinin ([Cunningham and O'Connor 1997]; [Yaron and Naider 1993]).

**Table 1:** **Effect of several solvents, cations, inhibitors, reducing agents and metal chelators on the activity of PepP of*Lactococcus lactis*ssp.*lactis*DSM 20481**

	**Substance**	**Concentration**	**Activity**^**1**^**[%]**	**Substance**	**Concentration**	**Activity**^**1**^**[%]**
Solvents [% (v/v)]	Acetone	10	79	DMSO	10	51
	EtOH		52			
Cations^2^ [mM]	Co^2+^	0.1	97	Sn^2+^	0.1	81
		1	86		1	4
		10	63		10	3
	Cu^2+^	0.1	73	Zn^2+^	0.001	100
		1	3		0.01	59
		10	3		0.1	4
	Mg^2+^	0.1	97			
		1	89			
		10	88			
Reagents [mM]	*β*-mercaptoethanol^2^	0.1	100	Pepstatin A^3^	0.001	113
		1	97		0.01	111
		10	106		0.1	111
	DTT^2^	0.1	99	1,10- phenanthroline^4^	0.1	103
		1	84		1	96
		10	51		10	53
	EDTA^2^	0.1	105		30	13
		1	102	PMSF^5^	0.1	120
		10	100		1	114
		30	1		10	10
	Imidazole^2^	0.1	112			
		1	107			
		10	109			

^1^For each enzyme activity test an individual 100 % value was determined in relation to the corresponding solvent.

^2^dissolved in H_2_O_dd_; ^3^dissolved in DMSO; ^4^dissolved in Acetone; ^5^dissolved in EtOH.

n = 3; all standard deviations < 5 %.

Due to the unique contribution of proline residues to protein hydrolysate bitterness, much research has focused on the application of proline-specific exopeptidases in hydrolysate debittering strategies due to the general inability of most general aminopeptidases to hydrolyze the imino bond ([FitzGerald and O'Cuinn 2006]). PepP from lactococcoal strains may contribute to the abolition of bitterness during cheese ripening when peptide degradation is involved during fermentation ([McDonnell et al. 1997]). Debittering of a tryptic hydrolysate of β-casein has been described using purified general and proline-specific aminopeptidases from *Lactococcus lactis* ssp. *cremoris* AM2 ([Bouchier et al. 2001]). Thus, PepP has industrial potential based on its application to the enzymatic debittering of proline-rich peptide mixtures in foods. In addition to native enzyme production and purification, recombinant DNA technology can be used to express large amounts of interesting enzymes ([Oliveira et al. 2011]). This possibility greatly expands the range of potential applications for enzymes (e.g., β-galactosidases) and their economically effective utilization in industrial processes ([Oliveira et al. 2011]).

PepP activity has previously been determined using different methods, such as a modified colorimetric ninhydrin method ([Doi et al. 1981]), various spectrophotometric assays employing other peptidases as auxiliary enzymes (proline iminopeptidase; EC 3.4.11.5 or dipeptidyl peptidase IV; EC 3.4.14.5) and substrates such as Gly-Pro-*p*NA or Gly-Pro-Pro-*p*NA ([Yoshimoto et al. 1988b]; [Lasch et al. 1988]). Furthermore, fluorescence-based assays have been employed to determine PepP activity using different synthesized fluorogenic substrates ([Fleminger et al. 1982]; [Holtzman et al. 1987]; [Hawthorne et al. 1997]; [Stöckel-Maschek et al. 2003]). However, all of these assays have different disadvantages ([Yoshimoto et al. 1988b]; [Doi et al. 1981]) with respect to accurately determining PepP activity.

In this study, we cloned and sequenced the genes encoding PepP from *Lactococcus lactis* ssp. *lactis* DSM 20481 and *Lactobacillus plantarum* NC8. Furthermore, we expressed lactococcal PepP for the first time in *E. coli*, and following automated purification, we characterized lactococcal PepP and developed a new gas chromatographic assay for accurate PepP enzyme activity determination and a novel specific activity staining method for PepP for use in native-PAGE analysis.

## Materials and methods

### Materials

All chemicals were obtained from Sigma-Aldrich Chemie GmbH (Schnelldorf, Germany), Roth (Karlsruhe, Germany), AppliChem GmbH (Darmstadt, Germany) or Merck AG (Darmstadt, Germany) and were of analytical-grade purity. Oligonucleotides were obtained via biomers.net GmbH (Ulm, Germany). Kits and enzymes for the molecular biological work were obtained from Qiagen (Hilden, Germany), Fermentas GmbH (St. Leon-Rot, Germany), New England Biolabs GmbH (Frankfurt am Main, Germany) or Roche (Mannheim, Germany). Standard peptides were obtained from Bachem AG (Bubendorf, Switzerland). Nucleotide sequence analyses were carried out by SRD – Scientific Research and Development GmbH (Bad Homburg, Germany). Chromatography materials (BioFox 40 IDA_low_) for protein purification as well as the Bioline chromatography system were provided by Wissenschaftliche Gerätebau Dr. Ing. Herbert Knauer GmbH (Berlin, Germany). Bioreactor cultivation was performed using the Multifors system (Infors AG, Bottmingen/Basel, Switzerland). For polyacrylamide gel electrophoresis, the MINI-PROTEAN system (Bio-Rad Laboratories GmbH, München, Germany) was employed.

### Bacterial strains and culture conditions

The *Lactococcus lactis* ssp. *lactis* DSM 20481 strain was cultivated in Medium 53 [glucose (5 g L^-1^), casein peptone, tryptic digest (10 g L^-1^), yeast extract (5 g L^-1^) and NaCl (5 g L^-1^)] under shaking at 30°C. The *Lactobacillus plantarum* NC8 strain was cultivated in de Man, Rogosa and Sharpe (MRS) medium [glucose (20 g L^-1^), casein peptone, tryptic digest (10 g L^-1^), meat extract (10 g L^-1^), yeast extract (5 g L^-1^), Na-acetate (5 g L^-1^), dipotassium hydrogen phosphate (2 g L^-1^), diammonium hydrogen citrate (2 g L^-1^), Tween 80 (1 g L^-1^), magnesium sulfate heptahydrate (0.2 g L^-1^) and manganese sulfate monohydrate (0.05 g L^-1^)] under shaking at 37°C.

*E. coli* DH5α (Invitrogen, Carlsbad, USA) and *E. coli* BL21 (DE3) (Novagen, Madison, USA) were used as the hosts for cloned PCR products and T7 expression work, respectively. Standard protocols were employed for the preparation and transformation of competent *E. coli* cells with plasmid DNA via heat shock ([Sambrook and Russell 2001]). Cells were cultivated in Luria Bertani (LB) medium [yeast extract (5 g L^-1^), peptone (10 g L^-1^) and NaCl (5 g L^-1^)] supplemented with the appropriate antibiotic [ampicillin (100 μg mL^-1^)] and agar (15 g L^-1^) for agar plates. Unless otherwise stated, all cultures were grown under shaking at 37°C.

### Cloning, construction of expression vectors and sequencing of *pepP*

Total genomic DNA from either *Lactococcus lactis* or *Lactobacillus plantarum* was extracted using the phenol/chloroform method. Briefly, 5 mL of an overnight culture was centrifuged (10 min, 3,000 x *g*), resuspended in suspension buffer (400 μL; 25 mM Tris/HCl, 50 mM glucose and 10 mM EDTA, pH 8.0) containing lysozyme (1 ng mL^-1^) and mutanolysin (50 U mL^-1^) and incubated for 45 min at 37°C. After the addition of 10% (w/v) SDS (40 μL), a further incubation (15 min, 65°C) was carried out. A second centrifugation step (10 min, 17,000 x *g*) was performed after the addition of phenol/chloroform (500 μL). The supernatant was gently mixed with 3 M Na-acetate [10% (v/v)], ice-cold isopropanol [100% (v/v)] and centrifuged (5 min, 17,000 x *g*) for DNA precipitation. The DNA was washed with ethanol [500 μL, 70% (v/v)] and resuspended in elution buffer (50 μL, Qiagen) containing RNase (20 μg mL^-1^).

For the amplification of the *pepP* gene of *Lactococcus lactis* ssp. *lactis* DSM 20481 (*pepP*-Lc), the primers *NdeI*_*pepP*-Lc_for (5’-AGGAGAATAAACATATGAGAATTGAAAAATTAAAAG-3’) and *XhoI*_*pepP*-Lc_rev (5’-ACTAATCTCGAGAATAACGATAAGCTCTTTTG-3’), were employed based on the nucleotide sequence of *pepP* from *Lactococcus lactis* subsp. *lactis* IL1403 (EMBL: AAK04789) available in the UniProt data base (UniProt ID: Q9CHN7). For the *pepP* gene of *Lactobacillus plantarum* NC8 (*pepP*-Lb), the primers *NdeI*_*pepP*-Lb_for (5’-GTGACGAACATATGAGTCGAGTTGAACGGTTAC-3’) and *XhoI*_*pepP*-Lb_rev (5’-ACTAATCTCGAGTAAAATCAGTAAATCACGAGTTGC-3’) based on the nucleotide sequence of the *pepP* gene from *Lactobacillus plantarum* WCFS1 (EMBL: CCC78909) available in the UniProt data base (UniProt ID: F9UNX0) were used in combination with HotStar HiFidelity polymerase (Qiagen) according to the instructions of the manufacturer. Amplification of the *pepP* genes was carried out as follows: a preliminary denaturation was performed at 95°C for 5 min, followed by 12 cycles of denaturation (15 s at 95°C), annealing (1 min at 52°C for *pepP*-Lc or at 60°C for *pepP*-Lb; both reduced by 1°C per cycle) and extension (2 min at 72°C), with a subsequent 35 cycles of denaturation (15 s at 95°C), annealing (1 min at 50°C for *pepP*-Lc or 55°C for *pepP*-Lb) and extension (2 min at 72°C), and a final extension was then performed at 72°C for 10 min.

The amplified *pepP* gene PCR products were purified (QIAquick Gel Extraction Kit) after electrophoresis in an agarose gel [1 % (w/v)]. The purified PCR products and the expression vector [pET20b (+)] were digested with the *NdeI* and *XhoI* restriction enzymes and purified as described above. Ligations were performed according to manufacturer’s (Promega) protocols using T4-ligase (Roche). For nucleotide sequence analysis (SRD), a plasmid mini-preparation (Fermentas) was carried out after overnight culture of *E. coli* DH5α transformed with the constructed expression vector pET20b (+)_*pepP-*Lc or pET20b (+)_*pepP-*Lb. Database searches were performed online with the programs blastn and blastp provided by the BLAST server ([Altschul et al. 1990]; [Gish and States 1993]). Blastp was used to align the amino acid sequences. All parameters were set at their default values.

### Expression of recombinant PepP in *E. coli* BL21 (DE3)

Transformed *E. coli* BL21 (DE3) strains were grown in 2xYT medium [yeast extract (10 g L^-1^), tryptone (16 g L^-1^) and NaCl (10 g L^-1^)] containing glucose (15 g L^-1^)] supplemented with ampicillin (100 μg mL^-1^). Precultures were incubated at 20°C on a rotary shaker. The first precultures were cultivated for 24 h and the second for 19 h. The main cultures (800 mL) were performed in a bioreactor parallel system (Multifors) and inoculated with 10% (v/v) preculture, each. The pH (6.5) was regulated using 3 M NaOH and 1 M H_3_PO_4_. The cultures were gas flushed (1 vvm) with air, and the pO_2_ was maintained over 30% by stirring at 500 rpm. The temperature was held constant at 16°C, and the optical density (OD_600 nm_) was determined with a spectral photometer at 600 nm. As the OD_600_ reached 5, the cells were induced by addition of 0.5 mM IPTG. The cultures were stopped after 60 h of cultivation. Glucose concentrations were analyzed using a photometric assay at 340 nm with the HK/G6P-DH-system (Megazyme International Ireland, Co. Wicklow, Ireland) in microtiter plates, based on the manufacturer’s protocol for the D-glucose/D-fructose test kit (R-Biopharm AG, Darmstadt, Germany; product code 10 139 106 035). For determination of cell dry weights (cdw), samples (1 mL) were first centrifuged (8,000 x *g*, 5 min, 4°C), and the cell pellets were resuspended in saline (1 mL) and centrifuged again. The obtained cell pellets were dried using a rotation vacuum concentrator (RVC 2–33 IR, Martin Christ Gefriertrocknungsanlagen GmbH, Osterode am Harz, Germany) for 6 h to 8 h (10 mbar, 40°C) until the weight was constant. For expression analyzes during cultivations 10 mL cell suspensions were centrifuged (8,000 x *g*, 5 min, 4°C), and the pellets were washed with saline and stored at −20°C. Crude cell extracts were prepared following suspension in 50 mM Tris/HCl buffer (pH 7.0) by sonification and centrifugation (8,000 x *g*, 5 min, 4°C). After culturing, the cells were harvested by centrifugation (8,000 x *g*, 15 min, 4°C), washed with saline and stored at −20°C.

### Automated purification of PepP

For protein purification, 15% (w/v) cell suspensions in 50 mM Tris/HCl buffer (pH 8.0) containing 500 mM NaCl were prepared and disrupted by sonification. The crude extracts were centrifuged (8,000 x *g*, 5 min, 4°C), and the resultant supernatants were passed through 0.45 μm filters. Purification and desalting were performed using an automated Bioline system (Knauer). The system was equipped with a pump (S 1000), DAD (S 2850), conductivity meter (S 2900) and a 6-port/3-channel manual injection valve. Chromatography columns were connected to the system via two 7-port/1-channel switching valves (see Additional file 1). Peak parking was accomplished with two 6-port/3-channel switching valves (see Additional file 1). Eluted proteins were collected with a fraction collector (Frac 3050). The fraction collector was cooled using a thermostat (Ministat 230, Huber, Germany) at 4°C. The Bioline chromatography system was controlled by the ChromGate Data System V.3.3.2.

The filtered cellular extract was applied to BioFox 40 IDA_low_ (Knauer) resin charged with Ni^2+^ [1 column volume (CV) = 11 mL]. The sample was loaded onto a column with a flow rate of 1 mL min^-1^, and the column was washed at a flow rate of 2 mL min^-1^ with 4 CV of 50 mM Tris/HCl buffer (pH 8.0) containing 500 mM NaCl and 10 mM imidazole to avoid nonspecific binding. PepP was eluted in a step in which the imidazole concentration was increased to 500 mM. The eluted protein fractions exhibiting PepP activity were injected into a Superloop^TM^ (GE Healthcare) from 39.75 – 46 min and desalted (50 mM Tris/HCl buffer, pH 7.0) automatically using HiPrep^TM^ 26/10 desalting columns (GE Healthcare). Desalted protein fractions showing PepP activity were collected in 5 mL volumes. The purified enzyme was finally stored at −80°C.

### Polyacrylamide gel electrophoresis (PAGE)

After cell disruption (sonification), samples were divided into soluble and insoluble fractions. These samples as well as the purified PepP [5 μg of protein each ([Bradford 1976])] were analyzed via sodium dodecyl sulfate (SDS)-PAGE [12.5%; ([Laemmli 1970])]. A standard molecular weight protein mixture was used as reference (NEB). Gels were stained for protein detection with Coomassie Brilliant Blue.

Native-PAGE (8%) was carried out on ice with the soluble samples before and after purification [unless otherwise stated, 5 μg protein was added per lane; ([Bradford 1976])]. A native standard molecular weight protein mixture obtained from SERVA Electrophoresis GmbH (Heidelberg, Germany) was used as reference. The gels were stained for protein detection with Coomassie Brilliant Blue.

To detect the specific activity of PepP, the staining method of [Lewis and Harris (1967]) was used, with some modifications. The native-PAGE gel was washed two times with 50 mL of Tris/HCl buffer (pH 7.0) containing 2 mM MnCl_2_. Then, 10 mL of solution A [50 mM Tris/HCl buffer (pH 7.0) containing 2 mM MnCl_2_, 2 mg *o*-dianisidine dihydrochloride, 5 mg LPP, 1 U L-amino acid oxidase (LAOX) and 240 U peroxidase (PER)] was mixed with 10 mL of solution B [50 mM Tris/HCl buffer (pH 7.0) containing 2 mM MnCl_2_ and 2% (w/v) agarose; temperature 55°C–60°C], thus producing solution C. The washed native-PAGE gel was covered with solution C and incubated at 37°C until an orange/brown-colored band appeared (see Additional file 2 for visualization scheme).

The activity staining was also used to verify the separation process of the recombinant PepP from the native PepP from *E. coli*. For these experiments, the host *E. coli* BL21 (DE)_pET20b (+) without the *pepP* gene (control) was purified using the same method as was employed for *E. coli* BL21 (DE)_pET20b (+)_*pepP*-Lc. In the control, PepP activity was only detected at 200 kDa in the crude cell extract as well as in the flow-through from the Ni^2+^ affinity column. The protein concentration loaded on the native-PAGE gel in this case was 10-fold higher compared to the recombinant PepP due to the low PepP activity in the control. Furthermore, it was approximately 5-fold longer before active bands appeared, and only weak bands at approximately 200 kDa could be detected (data not shown).

### Measurement of PepP activity using a gas chromatographic assay

The peptide L-Leu-L-Pro-L-Pro (LPP) was usually used as a substrate to determine PepP activity following standard procedures. The standard assay was carried out as follows: 5 μL of internal standard (ISTD; 5 mg mL^-1^ phenylalanine in H_2_O_dd_) and 5 μL of MnCl_2_ (150 mM in H_2_O_dd_) were added to 50 μL of enzyme solution (diluted in 50 mM Tris/HCl buffer, pH 7.0). After preincubation (5 min at 40°C), 10 μL of LPP solution (10 mg mL^-1^, dissolved in H_2_O_dd_) was added to the reaction mixture. The reaction was stopped after 5 to 10 min by addition of 5 μL of 1 M HCl. After centrifugation (8,000 x *g*, 5 min), 60 μL of the solution was transferred to a glass vial, and the amino acids and di- and tripeptides (substrates and products) were derivatized as described in the literature ([Husek 1991]), with some modifications. The sample was mixed with 80 μL of ethanol/pyridine (ratio 4:1) solution, and 10 μL of ethyl chloroformate (ECF) was added. Next, the solution was shaken (1,000 rpm) at room temperature for 5 min, and 100 μL of chloroform (containing 1% ECF) was then added, followed by shaking again for 5 min at 1,000 rpm. For phase separation, the reaction mixture was allowed to stand at room temperature without shaking for 5 min. Following this, 60 μL of the chloroform phase (the lower phase) was transferred to the GC vial and injected.

A Focus (Thermo Scientific, Germany) gas chromatography system equipped with an AS3000 auto sampler, flame ionization detector and TR-5 column (7 m x 0.32 mm x 0.25 μm; Thermo Scientific) was used for analysis of amino acids and di- and tripeptides. The initial column temperature was set to 80°C for 30 sec and then increased to 320°C at 35°C min^-1^ and held at 320°C for 5 min. The detector and injector were thermostated at 375°C and 290°C, respectively. Helium was used as the carrier gas at a flow rate of 3 mL min^-1^. The injection volume was 1 μL using splitless mode. One katal of PepP activity was defined as the amount of enzyme that released 1 mol of L-leucine (or another amino acid, depending on the substrate) per second. The specific activity was defined as the proteolytic activity per mg of protein used in this assay. The protein concentration was quantified using bovine serum albumin as a standard ([Bradford 1976]).

### Characterization of PepP

Characterization was carried out using purified PepP (see above).

#### Determination of enzyme kinetics

The kinetic parameters (*K*_*m*_ and *V*_*max*_) of PepP were determined with the substrates L-Leu-L-Pro-L-Pro (LPP), L-Ile-L-Pro-L-Pro (IPP) and L-Val-L-Pro-L-Pro (VPP). Standard conditions for PepP activity determination (see above) were used, except regarding the substrate concentration in assay. The applied substrate concentrations ranged from 0.085 mM to 12.2 mM depending on the substrate.

#### Effect of pH and temperature on the enzyme activity

To determine the pH profile of PepP, the activity was assayed under standard conditions (see above), except that the enzyme was diluted in the corresponding buffer. The pH value ranged from 5.0 to 8.5.

To assess the effect of temperature on enzyme activity, the activity was assayed under standard assay conditions (see above), except that the assay temperature was varied between 20°C and 70°C. To provide temperature stability, the enzyme solution was incubated at different temperatures ranging from 0°C to 50°C over a period of 15 days. The residual activity was subsequently measured under standard assay conditions (see above).

#### Effects of freezing and thawing

To assess the effect of freezing, aliquots of the enzyme solution were frozen at −80°C and analyzed over a period of 15 days using a standard PepP activity assay.

The influence of freeze cycles on the PepP enzyme activity was determined by freezing the enzyme solution at −80°C, then defrosting it, analyzing PepP activity, and freezing the solution again. A total of 6 freeze cycles were performed.

#### Effect of Mn^2+^ on enzyme activity

To determine the effect of different Mn^2+^ concentrations of PepP activity, standard conditions for PepP activity determination (see above) were applied, except that different concentrations of MnCl_2_ (0 mM to 14.28 mM in the assay) were tested.

#### Effects of inhibitors, cations, metal chelators, reducing agents and solvents on enzyme activity

The tested substances were dissolved or diluted in H_2_O_dd_, DMSO, acetone or ethanol, depending on the substance. The assay conditions were the same as under the standard protocol (see above). Different from the standard protocol, 7 μL of the test substance and 43 μL of enzyme solution were used instead of 50 μL of enzyme solution, and the preincubation time was from 5 min to 15 min. All metal ions were added as chlorides to prevent any influence of anions.

## Results

### Cloning and sequencing of the *pepP* genes of *Lactococcus lactis* ssp. *lactis* DSM 20481 and *Lactobacillus plantarum* NC8

Amplification of the *pepP* gene from the genome of *Lactococcus lactis* ssp. *lactis* DSM 20481 resulted in an ~ 1100-bp-long PCR product (*pepP*: 1059 bp). The fragment was cloned into the pET20b (+) expression vector, producing pET20b (+)_*pepP-*Lc. This vector was sequenced (see Additional file 3 for sequence) and used for expression in the *E. coli* BL21 (DE3). The new nucleotide sequence of *Lactococcus lactis* ssp. *lactis* DSM 20481 (accession number: JX155860) was compared (blastn) to other *pepP genes,* showing 99% identity to *Lactococcus lactis* ssp. *lactis* KF147, *Lactococcus lactis* ssp. *lactis* CV56 and *Lactococcus lactis* ssp. *lactis* IL1403. The identities shared with the *pepP* genes *of Lactococcus lactis* ssp. *cremoris* NZ9000 and *Lactococcus lactis* ssp. *cremoris* MG1363 were 86%. Alignment of the obtained amino acid sequence (blastp) to other PepP sequences showed 99% amino acid sequence identity to *Lactococcus lactis* ssp. *lactis* IL1403 (1 different amino acid) and 91% amino acid sequence identity to *Lactococcus lactis* ssp. *cremoris* MG1363 (32 different amino acids). The amplification of the *pepP gene* from the genome of *Lactobacillus plantarum* NC8, resulted in an ~ 1100-bp-long PCR product (*pepP*: 1062 bp), which was then treated as described above, producing pET20b (+)_*pepP-*Lb (see Additional file 4 for the sequence). The sequenced *pepP* gene of *Lb. plantarum* NC8 (current work) was 100% identical to the current published sequence of the *pepP* gene from *Lb. plantarum* NC8 (UniProt ID: H3P3J7). Furthermore, the nucleotide sequence was 100% identical to the putative *pepP* gene of *Lactobacillus plantarum* ssp. *plantarum* ST-III. Additionally, 99% identity of the newly obtained *pepP* gene was observed with *Lactobacillus plantarum* JDM1 and *Lactobacillus plantarum* WCFS1. Alignment of the amino acid sequence (blastp) to other PepP sequences from *Lactobacillus plantarum* strains with less than 100% nucleotide sequence identity showed 100% amino acid sequence identity for *Lactobacillus plantarum* JDM1 and 99% amino acid sequence identity for *Lactobacillus plantarum* WCFS1 (1 different amino acid).

### Recombinant expression of PepP in *E. coli*

**Figure 1:**
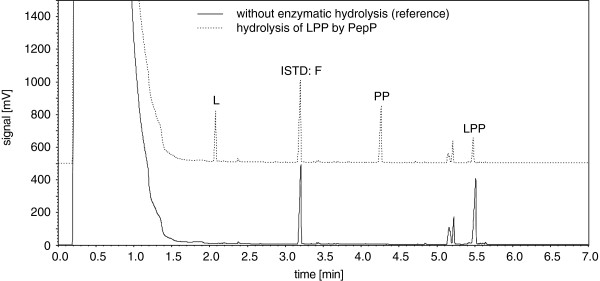
**GC-FID chromatograms before and after hydrolysis of LPP by PepP.** (L: L-leucine; ISTD: internal standard, F: L-phenylalanine; PP: L-prolyl-L-proline; LPP: L-leucine-L-prolyl-L-proline).

In preliminary shaking flasks experiments the recombinant expression of PepP from *Lactobacillus plantarum* NC8 as well as *Lactococcus lactis* ssp. *lactis* DSM 20481 was studied. The cultivation temperatures varied between 4°C and 37°C. At higher temperatures (30°C and 37°C), no active recombinant protein was detected in the soluble fractions after cell disruption, but a strong overexpression band was observed following SDS-PAGE analysis of the insoluble protein fraction (inclusion bodies; data not shown). Cultivation at lower temperatures (4°C and 20°C) resulted in a recombinant protein overexpression band in both the soluble and insoluble protein fractions after cell disruption (data not shown). Similar results were obtained using *E. coli* C41 (DE3) and *E. coli* C43 (DE3) as hosts for expression. The expression systems containing partial soluble PepP were tested for enzymatic function using a novel, specific PepP activity assay based on the release of L-leucine from the substrate LPP (Figure 1).

**Figure 2:**
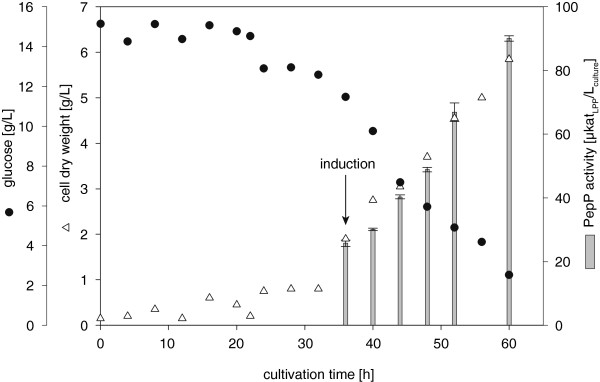
**Production of PepP from*Lactococcus lactis*ssp.*lactis*DSM 20481 in*E. coli*BL21 in a bioreactor.** (working volume: 800 mL; 2xYT medium; 16°C; induction with 0.5 mM IPTG).

To determine the background activity of the *E. coli* BL21 (DE3) expression host, *E. coli* BL21 (DE) transformed with pET20b (+) without the *pepP* gene (reference) was cultivated under the same conditions described above. The background PepP activity of the reference was approximately 1% of that detected in the *E. coli* BL21 (DE)_ pET20b (+)_*pepP-*Lc expression system (data not shown). Regarding the putative PepP from *Lactobacillus plantarum* NC8, using the expression system *E. coli* BL21 (DE)_ pET20b (+)_*pepP-*Lb, no increase of PepP activity compared to the reference was achieved (data not shown). Additionally, we cultivated *E. coli* BL21 (DE)_pET20b (+)_*pepP-*Lc in shaking flasks at 37°C and cooled the cultures to 4°C and 20°C prior induction. However, in these cases, no PepP expression was detected in the soluble protein fraction (data not shown). Therefore, all further cultivations were performed using a bioreactor at a constant temperature of 16°C, and induction (0.5 mM IPTG) was carried out at OD_600 nm_ = 5 (Figure 2). After 60 h of cultivation, an intracellular PepP activity of 90 μkat_LPP_ L_culture_^-1^ was achieved (for sample preparation, see Materials and Methods). It can be assumed that even higher activity yields could be possible under longer cultivations times because glucose was not fully consumed during this cultivation.

### Automated purification of recombinant PepP and molecular mass estimation

**Figure 3:**
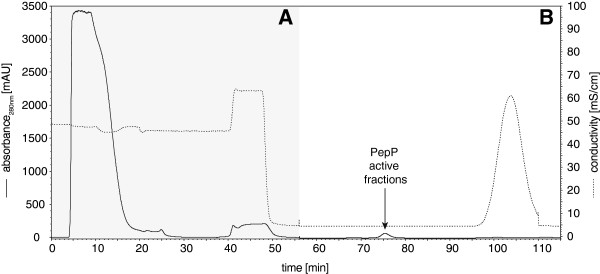
**Automated purification of recombinant PepP using (A) Ni**^**2+**^**-affinity chromatography and (B) two HiPrep**^**TM**^**26/10 columns (desalting).**

PepP was purified automatically using a Ni^2+^ affinity column, with subsequent desalting via two HiPrep^TM^ 26/10 columns (Figure 3). Therefore, two 7-port/1-channel switching valves were employed for connection of the chromatography columns to the chromatography system. For “peak parking” and automatic injection of the parked proteins, two 6-port/3-channel switching valves were used (see Additional file 1). PepP was first purified using a Ni^2+^ affinity column operated with 50 mM Tris/HCl buffer (pH 8.0) containing 500 mM NaCl and 10 mM imidazole. PepP was eluted in a step in which the imidazole concentration was increased to 500 mM (after ~ 40 min) in the same buffer. Subsequently, the purified PepP was automatically desalted in 50 mM Tris/HCl buffer (pH 7.0). Finally, an enzyme activity yield of 82% and a purification factor of 22.9 (specific activity: 3682 nkat_LPP_ mg_protein_^-1^) were determined for PepP.

**Figure 4:**
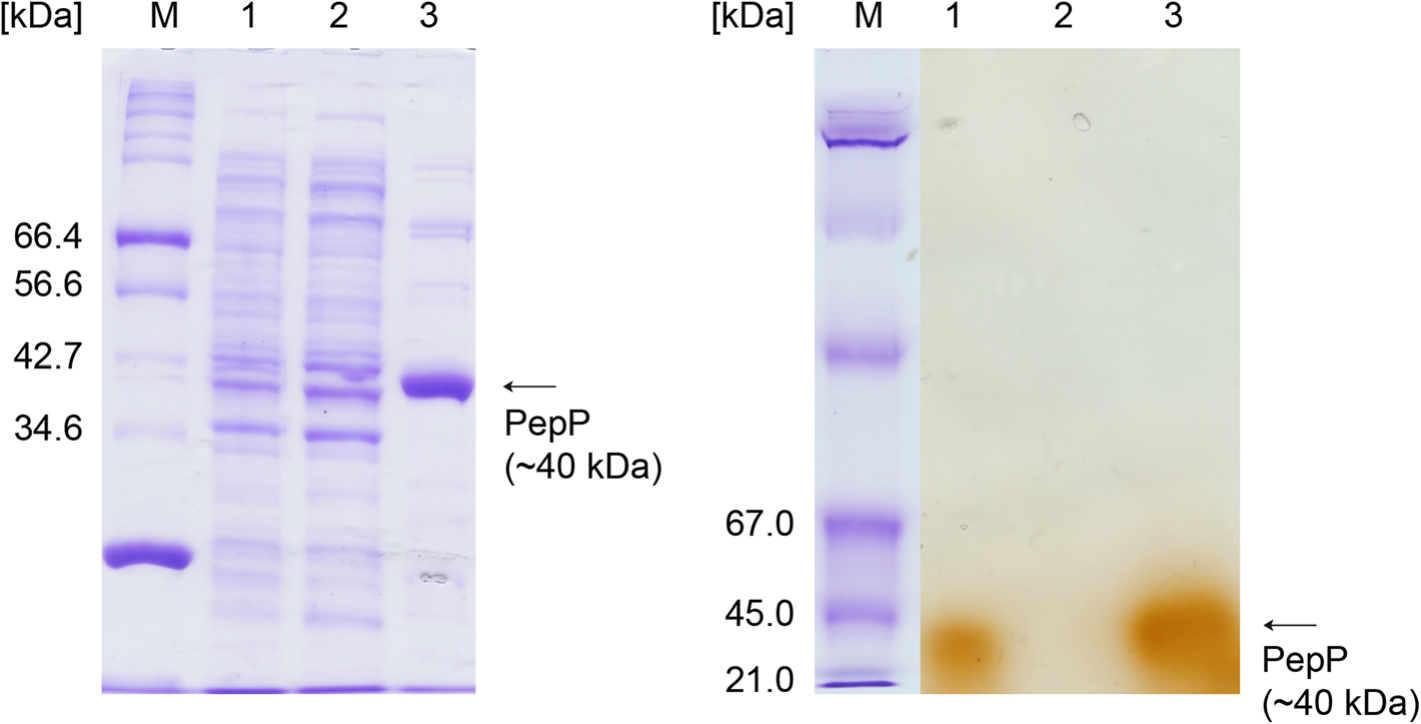
**SDS-PAGE (left; Coomassie stained) and native-PAGE (right; lanes 1 – 3 active stained) analyses of recombinant PepP produced in*E. coli*BL21 (DE3) during purification.** (M: Molecular weight marker; lane 1: crude cell extract before purification; lane 2: pooled flow-through fractions: lane 3: pooled purified fractions after desalting).

SDS-PAGE analysis of the samples during cultivation showed an overexpression band at approximately 40 kDa for PepP (data not shown). Thus, PepP could be determined in both the soluble and insoluble fractions (inclusion bodies; data not shown). SDS-PAGE analysis after purification showed that PepP bound strongly to the affinity chromatography material (see Figure 4; SDS-PAGE, lane 2), as no significant amount of the His-tagged recombinant PepP was eluted in the flow-through fractions.

Performing native-PAGE together with Coomassie and specific activity staining of PepP after purification revealed bands of approximately 40 kDa (see arrows in Figure 4). These results indicated that the purified PepP was a monomer with a molecular mass of approximately 40 kDa (calculated based on the amino acid sequence including a His-tag: 40.6 kDa).

Staining to detect the specific activity of PepP was also employed to evaluate the separation of the native *E. coli* PepP [see above; homotetramer, 200 kDa; [Yoshimoto et al. 1988a] from the recombinant produced PepP during the purification process (see Materials and methods). The recombinant purified PepP contained no activity stained band at 200 kDa. Thus, it can be assumed that no native PepP from the *E. coli* host or other LPP hydrolyzing enzymes were present in the purified, recombinant enzyme preparation and that all of the determined enzyme characteristics were associated with the new, recombinant PepP produced from *Lactococcus lactis* ssp. *lactis* DSM 20481.

### Characterization of purified PepP

#### Temperature and pH optimum

**Figure 5:**
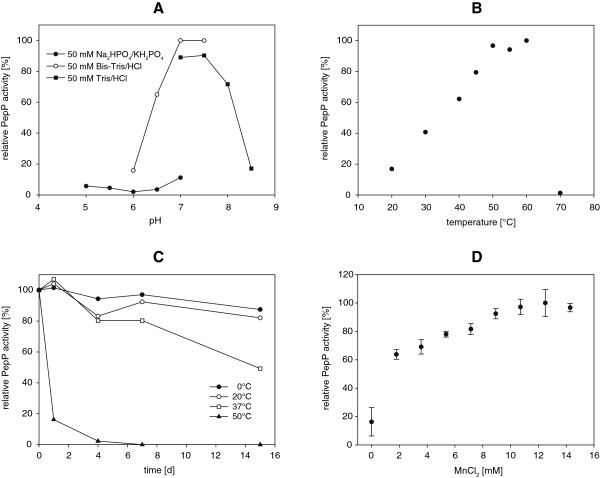
**Characterization of recombinant purified PepP from*Lactococcus lactis*ssp.*lactis*DSM 20481.** (**A:** pH profile; **B:** temperature profile; **C:** temperature stability; **D:** influence of MnCl_2_; n = 3, standard deviations < 5 % are not shown).

The pH optimum was assayed by measuring enzyme activities at different pH levels (Figure 5A). PepP showed the highest activity between pH 7 and 7.5 when using 50 mM Bis-Tris/HCl buffer. A strong negative buffer effect was detected when using 50 mM Na_2_HPO_4_/KH_2_PO_4_ (pH 5.0 to 7.0).

To investigate the optimal reaction temperatures for PepP, the hydrolysis of LPP was assayed at different temperatures (Figure 5B). PepP showed the highest activity between 50°C and 60°C and was inactivated at 70°C.

The temperature stability of PepP was determined by preincubating purified PepP in 50 mM Tris/HCl buffer, pH 7.0, at different temperatures (0, 20, 37 and 50°C). The highest temperature stability of PepP (Figure 5C) was found at 0°C and 20°C (87% residual activity and 82% residual activity after 15 days of incubation, respectively). Approximately 49% residual PepP activity was detected at 37°C after 15 days. At the temperature optimum for PepP (50°C), the residual activity after one day was 16%, and no activity could be observed after 7 days of incubation at this temperature.

#### Influence of freezing and thawing

Purified PepP was stored at −80°C over a period of 15 days. During this time, aliquoted samples were checked for residual activities and no significant inactivation of PepP was observed (97% residual activity; data not shown). Additionally, the influence of 6 alternating cycles of freezing and thawing was observed. The residual activity of PepP was 87% after these cycles (data not shown).

#### Effect of Mn^2+^-ions on PepP enzyme activity

To investigate the optimal Mn^2+^ concentration for PepP activity, MnCl_2_ was used in different concentrations, with the final concentrations ranging from 0 mM to 14.28 mM (Figure 5D). The PepP activity obtained without added MnCl_2_ was 3.9-fold lower compared to 1.79 mM MnCl_2_. MnCl_2_ exerted a favorable effect on PepP activity up to a final MnCl_2_ concentration of 8.92 mM. Between 8.92 mM and 14.28 mM, no additional significant increase of PepP activity was detected (approximately 6-fold increases compared to the reference for all of these concentrations).

#### Enzyme kinetics

**Figure 6:**
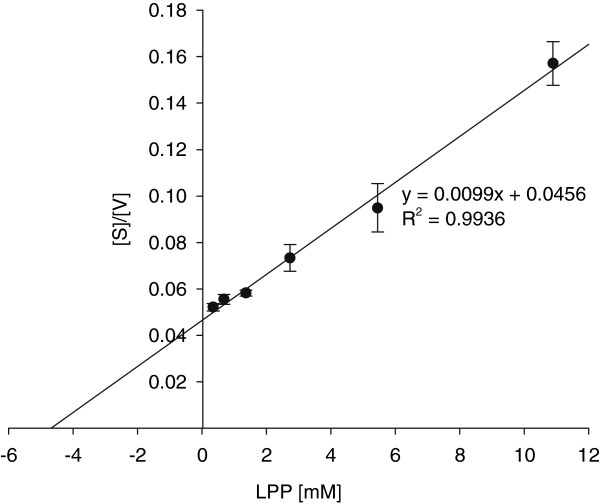
**Calculation of*K***_***m***_**and*V***_***max***_**for PepP via a Hanes plot using LPP as a substrate.**

The kinetics were determined for PepP using LPP, IPP and VPP as peptide substrates. The *K*_*m*_ values and *V*_*max*_ values were calculated by linearization following Hanes (for an example, see Figure 6). The *K*_*m*_ and *V*_*max*_ values calculated for PepP when using LPP as substrate were 4.7 mM and 101 nkat mL^-1^, respectively. The kinetics of PepP with IPP and VPP as substrates were found to present values of 2.5 mM and 13.6 mM for *K*_*m*_ and 74 nkat mL^-1^ and 145 nkat mL^-1^ for *V*_*max*_, respectively.

#### Effect of metal ions on enzyme activity

PepP was observed to be inhibited by several different divalent cations (see Table 1), despite fact that the standard assay was performed in the presence of an optimal MnCl_2_ concentration. Almost complete inhibition of enzyme activity was caused by 10 mM CuCl_2_, 1 mM SnCl_2_ or 10 mM ZnCl_2_. Additionally, marked inhibition was observed in the presence of 10 mM CoCl_2_ (63% residual activity), 0.1 mM CuCl_2_ (73% residual activity) and 0.01 mM ZnCl_2_ (59% residual activity). A concentration of 1 mM CoCl_2_ reduced the PepP activity to 86%. The divalent cation Mg^2+^ (from MgCl_2_)was associated with only weak inhibition up to a concentration of 10 mM (88% residual activity).

#### Effect of solvents, inhibitors, reducing agents and metal chelators on enzyme activity

The influences of several solvents and other agents on the PepP activity are also shown in Table 1. The PepP enzyme activity was reduced by the half when using 10% (v/v) ethanol or DMSO. Acetone [10% (v/v)] reduced the PepP activity to 79%. Almost no inhibition of PepP activity was observed under up to 1 mM PMSF (phenylmethylsulfonyl fluoride), which is a specific inhibitor of serine peptidases. Surprisingly, 10 mM PMSF reduced the PepP activity to 10% residual activity. In experiments using Pepstatin A, a specific carboxy peptidase inhibitor, no inhibition of PepP activity was detected. Preincubation of the enzyme with the reducing agent *β*-mercaptoethanol had no effect on the activity of PepP. However, the PepP activity decreased approximately 50% using 10 mM DTT (dithiothreitol). Imidazole did not lead to any inhibition of PepP activity at a concentration of up to 10 mM. Metal complexing reagents such as EDTA and 1,10-phenanthroline produced no inactivation up to 10 mM. However, at higher concentrations (30 mM), total inhibition was observed under EDTA treatment, and strong inhibition (13% residual activity) was detected under 1,10-phenanthrolinetreatment. The reason for this pattern was the high content of MnCl_2_ (10.71 mM) in the activity buffer. These results indicated that PepP is a metallopeptidase.

## Discussion

The classical assay methods for PepP activity determination present some problems ([Yoshimoto et al. 1988b]; [McDonnell et al. 1997]; [Lasch et al. 1988]; [Fleminger et al. 1982]; [Holtzman et al. 1987]; [Hawthorne et al. 1997]; [Stöckel-Maschek et al. 2003]). A precise determination of PepP activity using ninhydrin is nearly impossible because crude extracts contain large amounts of ninhydrin-positive substances ([Yoshimoto et al. 1988b]). It is also difficult to measure an initial reaction velocity via fluorometric methods ([Doi et al. 1981]). The method employing Gly-Pro-*p*NA coupled with proline iminopeptidase ([Yoshimoto et al. 1988b]) can be used for routine assays, but not for screening the PepP enzyme activity in crude extracts from lactic acid bacteria without further pre-treatment because of the existence of parallel PepX activity (X-prolyl dipeptidyl aminopeptidase; EC 3.4.14.11), which is common in lactic acid bacteria. Our novel assay method using gas chromatography for detection of the release of L-leucine during hydrolysis of the substrate (LPP) could be used for routine analysis as well as for screening experiments. Even when free amino acids are present in the crude extract, or other aminopeptidases are present, such as PepX, which did not hydrolyze Xaa-Pro-Pro peptide sequences, our GC assay is applicable and specific for PepP activity.

The gene (EMBL: CCC78909) of *Lactobacillus plantarum* WCFS1 encodes Xaa-Pro aminopeptidase (PepP). The gene encoding PepP in *Lactobacillus plantarum* NC8 showed 99% nucleotide and amino acid sequence identities (1 different amino acid) compared to *Lactobacillus plantarum* WCFS1. However, no PepP activity of *Lb. plantarum* NC8 was detected, even though overexpression was visible in SDS-PAGE gels.

In the literature, several lactococcus strains showing PepP activity have been reported ([Mars and Monnet 1995]; [McDonnell et al. 1997]; [Matos et al. 1998]). Therefore, we cloned the *pepP gene* of *Lactococcus lactis* ssp. *lactis* DSM 20481. To best of our knowledge, we are the first to express PepP from a lactococcal strain in *E. coli*. During our experiments, we found that expression at common temperatures (30°C and 37°C) resulted in insoluble recombinant PepP (inclusion bodies). When the cultivation temperature was decreased to 20°C (shaking flasks) or 16°C (bioreactor), the insolubility of PepP was partially overcome. Protein expression in *E. coli* at 15°C to 25°C is widely used to increase the solubility of recombinant proteins ([Song et al. 2012]). Here, it was demonstrated for all of the investigated recombinant proteins (*β*-mannanase, cellulase and lipase), which formed inclusion bodies when overexpressed at 15°C to 37°C, that they were expressed as soluble forms in *E. coli* when the temperature was lowered below 10°C. The reason for soluble protein production below 10°C was not clear. It has been discussed that the reason for this phenomenon may be the reduced growth rates at 6°C to 10°C ([Song et al. 2012]). Nevertheless, *E. coli* can still grow at 7.5 to 7.8°C, although its growth rate is drastically decreased ([Shaw et al. 1971]). We observed only slow growth of *E. coli* during the first 35 h of cultivation at 16°C, until exponential growth began. The maximal achieved PepP activity was 90 μkat_LPP_ L_culture_^-1^. After automated purification, a 700-fold higher enzyme activity of 73.8 μkat_LPP_ L_culture_^-1^ was achieved compared to the activity of 0.105 μkat_bradykinin_ L_culture_^-1^ for the native PepP produced and purified from *Lactococcus lactis* ssp*. lactis* NCDO 763 ([Mars and Monnet 1995]). Furthermore, the specific activity of the recombinant PepP that was produced and purified (3682 nkat_LPP_ mg^-1^) was approximately 20 % higher than that of the native produced and purified PepP (3073 nkat_bradykinin_ mg^-1^). Moreover, compared to the native PepP produced and purified from *Lactococcus lactis* ssp. *cremoris* AM2 (22.8 nkat_LPP_ mg^-1^; [McDonnell et al. 1997]), the specific PepP activity of the recombinant produced and purified PepP was 165-fold higher.

The observed molecular weight of PepP of ~40 kDa (monomer) agreed well with the values reported for PepP from *Lactococcus lactis* ssp. *cremoris* AM2 [40 kDa ([McDonnell et al. 1997])] and from *Lactococcus lactis* ssp. *lactis* NCDO 763 [43 kDa ([Mars and Monnet 1995])]. This weight is much lower than has been reported for *E. coli* PepP [homotetramer, 200 kDa ([Yoshimoto et al. 1988b])] or mammalian PepP [homodimer, 143 kDa ([Harbeck and Mentlein 1991])]. The enzyme examined in the present study appeared to be a metalloenzyme stimulated by Mn^2+^, similar to the PepPs from *Lactococcus lactis* ssp. *cremoris* AM2 ([McDonnell et al. 1997]) and *Lactococcus lactis* ssp. *lactis* NCDO 763 ([Mars and Monnet 1995]). Like the *Lactococcus lactis* enzyme ([Mars and Monnet 1995]), the present enzyme was inhibited by DTT, whereas *Lactococcus lactis* ssp. *cremoris* AM2 PepP is ([McDonnell et al. 1997]). In our study, we used the same substrate (LPP) as [McDonnell et al. (1997]), but a different detection method because quantification using the ninhydrin detection method was inappropriate in our case (data not shown). We determined a pH optimum for PepP between 7 and 7.5, while PepP from *Lactococcus lactis* ssp. *lactis* NCDO 763 ([Mars and Monnet 1995]) showed a pH optimum of 8, and the *Lactococcus lactis* ssp. *cremoris* AM2 PepP ([McDonnell et al. 1997]) displayed an optimum activity at pH 8.5. The determined *K*_*m*_ value for PepP when using LPP as substrate was 4.7 mM and, thus, was higher than that of *Lactococcus lactis* ssp. *cremoris* AM2 (0.9 mM_LPP_; [McDonnell et al. 1997]).

Aminopeptidase P may be involved in supplying proline in lactococcoal strains ([Mars and Monnet 1995]). Caseins, especially *β*-casein, can be hydrolyzed by proteinases and by aminopeptidases located on the cell envelope, releasing several sequences with Xaa-Pro-Pro N-termini, which cannot cleaved by lactococcal PepX ([Lloyd and Pritchard 1991]) but can be cleaved by PepP. Much research has focused on the application of proline-specific exopeptidases in hydrolysate debittering strategies due to the inability of most general aminopeptidases to hydrolyze the imino bond ([FitzGerald and O'Cuinn 2006]). Highly significant reductions in casein hydrolysate bitterness can be achieved using X-prolyl-dipeptidyl aminopeptidase (PepX), which releases amino acyl proline residues from the N-terminus, in conjunction with the activity of a general aminopeptidase (PepN; EC 3.4.11.2) ([FitzGerald and O'Cuinn 2006]). PepX together with PepP can mediate the hydrolysis of proline-rich substrates, i.e., those containing single and consecutive prolines ([Bouchier et al. 1999]; [O'Cuinn et al. 1999]).

Recombinant production of PepP provides the opportunity for further investigations regarding the debittering process of protein hydrolysates in large-scale applications. Thus, industrial application of PepP might be desirable in the future.

## Competing interests

The authors declare that they have no competing interests.

## Supplementary Material

Additional file 1 **Scheme of the automated purification.** This file contains the general flow scheme of the automated purification for proteins. Click here for file

Additional file 2 **Scheme of active staining.** This file contains the visualization scheme for activity staining of PepP. Click here for file

Additional file 3 **Sequences of*pepP*/PepP.** This file contains the nucleotide sequence of the *pepP* gene and the translated amino acid sequence from *Lactococcus lactis* ssp. *lactis* DSM 20481. Click here for file

Additional file 4 **Sequences of*pepP*/PepP.** This file contains the nucleotide sequence of the *pepP* gene and the translated amino acid sequence from *Lactobacillus plantarum* NC8. (PDF 219 kb) Click here for file
