# Supplementary material for: Production, active staining and gas chromatography assay analysis of recombinant aminopeptidase P from Lactococcus lactis ssp. lactis DSM 20481
Source: AMB Express. 2012 Aug 1;2:39. doi: 10.1186/2191-0855-2-39 (PMC3418211; doi:10.1186/2191-0855-2-39)
Supplement: Additional file 1 — Scheme of the automated purification. This file contains the general flow scheme of the automated purification for proteins. [file 2191-0855-2-39-S1.pdf]

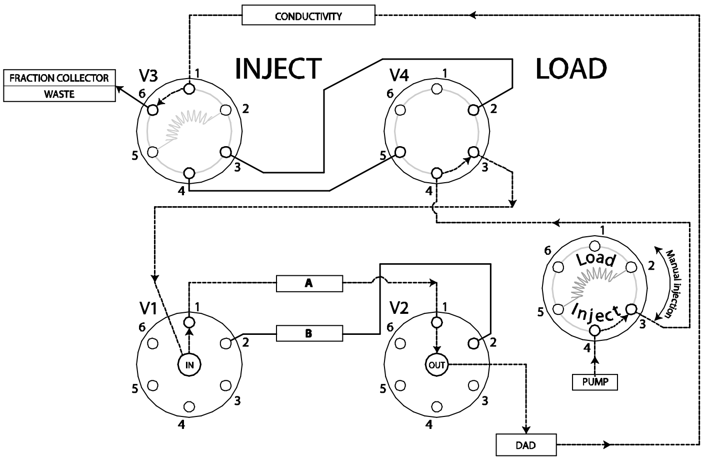

a) Initial position

In the initial position is the eluting protein directed to the waste or to the fraction collector (valve 3: INJECT; valve 4: LOAD)

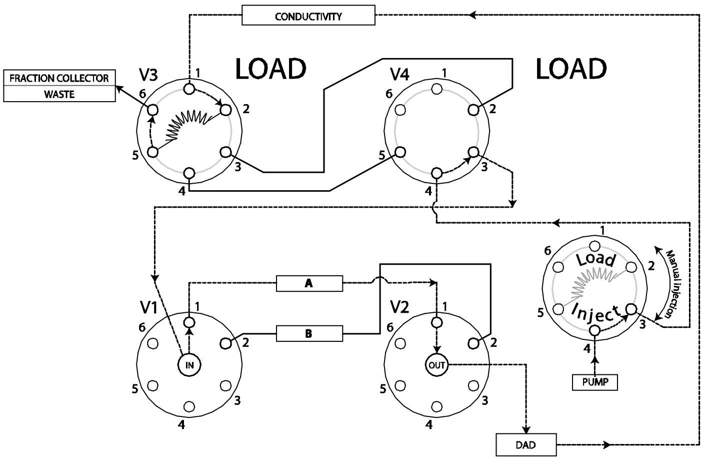

b) Peak parking

In the “peak parking modus” is eluting protein directed to the Superloop via valve 3 (valve 3: LOAD; valve 4: LOAD)

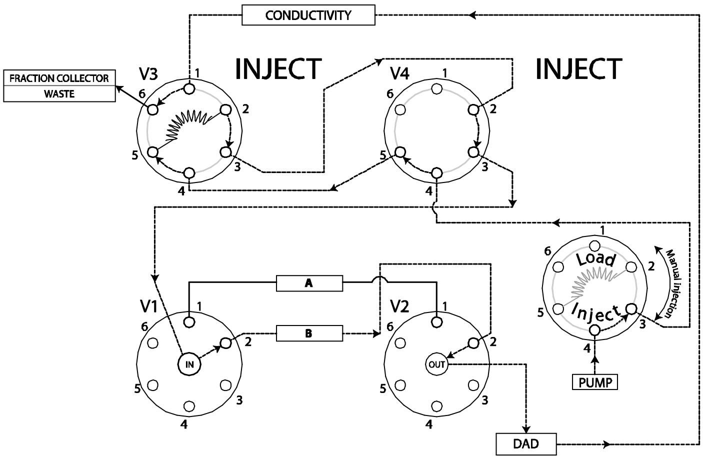

c) Automated injection

Automated injection of “parked” proteins from the Superloop^{TM} to chromatography system was performed via valves 3 and 4. Buffer is directed over valve 4 to valve 3 into the Superloop^{TM} and following is parked protein over valve 4 subjected to the next chromatography column (valve 3: INJECT; valve 4: INJECT)

Additional file 1. General flow scheme of the automated purification for proteins

(a: valve position for standard chromatography, b: valve position for peak parking, c: valve position to injection previously parked protein)
