# Supplementary figures and images for: Production, active staining and gas chromatography assay analysis of recombinant aminopeptidase P from Lactococcus lactis ssp. lactis DSM 20481
Source: AMB Express. 2012 Aug 1;2:39. doi: 10.1186/2191-0855-2-39 (PMC3418211; doi:10.1186/2191-0855-2-39)

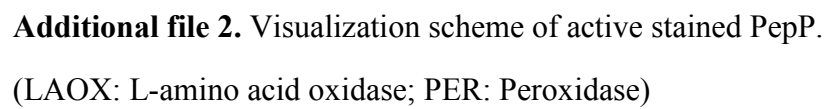

(LAOX: L-amino acid oxidase; PER: Peroxidase)

Supplement: Additional file 2 — Scheme of active staining. This file contains the visualization scheme for activity staining of PepP. [file 2191-0855-2-39-S2.pdf]
