# Supplementary material for: Production, active staining and gas chromatography assay analysis of recombinant aminopeptidase P from Lactococcus lactis ssp. lactis DSM 20481
Source: AMB Express. 2012 Aug 1;2:39. doi: 10.1186/2191-0855-2-39 (PMC3418211; doi:10.1186/2191-0855-2-39)
Supplement: Additional file 3 — Sequences ofpepP/PepP. This file contains the nucleotide sequence of the pepP gene and the translated amino acid sequence from Lactococcus lactis ssp. lactis DSM 20481. [file 2191-0855-2-39-S3.pdf]

NdeI

```
GGA GAT ATA CAT ATG AGA ATT GAA AAA TTA AAA GCA AAA ATG CTG ACA GAA AAT ATT GAT  < 60
                M   R   I   E   K   L   K   A   K   M   L   T   E   N   I   D
AGT TTA TTG ATT ACT GAT ATG AAG AAT ATC TTT TAT CTG ACA GGA TTT TCT GGA ACC GCG  < 120
S   L   L   I   T   D   M   K   N   I   F   Y   L   T   G   F   S   G   T   A
GGC ACA GTT TTT TTG ACA GCG AAG CGA AAT ATT TTT ATG ACA GAC AGC CGT TAT AGT GAA  < 180
G   T   V   F   L   T   A   K   R   N   I   F   M   T   D   S   R   Y   S   E
ATG GCG CGT GGA TTG ATT AGT GAT TTT GAA ATT ATC GAA ACA AGA GAT CCA ATA AGT TTA  < 240
M   A   R   G   L   I   S   D   F   E   I   I   E   T   R   D   P   I   S   L
CTG ACA GAC CTG TCA GCA AGT GAA TCA ATT AAA AAT ATA GCT TTT GAA GAA ACA GTT GAC  < 300
L   T   D   L   S   A   S   E   S   I   K   N   I   A   F   E   E   T   V   D
TAT GCT TTC TTT AAA AGA CTT TTT GAC GCC ACT CCA GGA TTA GAA CTT TTA GCC ACA AGT  < 360
Y   A   F   F   K   R   L   F   D   A   T   P   G   L   E   L   L   A   T   S
AAT TTT GTT TTG GAA TTG CGC CAA TTT AAA GAT GAC ACA GAG ATT GAT TTG ATA AAA AAA  < 420
N   F   V   L   E   L   R   Q   F   K   D   D   T   E   I   D   L   I   K   K
GCC TGT GCA ATT GCT GAT GAA GCG TTT ATG TCA GCT TTA AAA TTC ATT GAG CCG GGC CGA  < 480
A   C   A   I   A   D   E   A   F   M   S   A   L   K   F   I   E   P   G   R
ACT GAA ATT GAA GTT GCT AAT TTT CTT GAT TTC AAA ATG CGT GAT TTA GAG GCG AGC GGA  < 540
T   E   I   E   V   A   N   F   L   D   F   K   M   R   D   L   E   A   S   G
ATT TCC TTC GAA ACC ATT GTT GCC TCA GGC AAG AGA AGT AGC TTG CCT CAT GGC GTT GCG  < 600
I   S   F   E   T   I   V   A   S   G   K   R   S   S   L   P   H   G   V   A
ACC AGT AAA ATG ATT CAA TTT GGT GAT CCA GTG ACC ATT GAT TTT GGT TGT TAT TAT GAA  < 660
T   S   K   M   I   Q   F   G   D   P   V   T   I   D   F   G   C   Y   Y   E
CAT TAT GCT AGC GAT ATG ACC AGA ACA ATT TTC GTG GGT TCT GTA GAC GAC AAG ATG CGA  < 720
H   Y   A   S   D   M   T   R   T   I   F   V   G   S   V   D   D   K   M   R
ACA ATT TAT GAA ACC GTG CGT AAG GCT AAT GAA GCA CTT ATC AAA GAA GTC AAA GCG GGA  < 780
T   I   Y   E   T   V   R   K   A   N   E   A   L   I   K   E   V   K   A   G
ATG ACT TAT GCC GAA TAT GAT AAG GTT CCG CGT ACC GTC ATT GAA GAA GCC AAT TTT GGT  < 840
M   T   Y   A   E   Y   D   K   V   P   R   T   V   I   E   E   A   N   F   G
CAG TAT TTT ACG CAT GGA ATT GGA CAT GGA CTT GGT TTA GAT GTT CAT GAA ATT CCT TAT  < 900
Q   Y   F   T   H   G   I   G   H   G   L   G   L   D   V   H   E   I   P   Y
TTT AAT CAA TCA ATG ACA GAA AAT CAC TTG GAA GCT GGG ATG GTT ATT ACA GAT GAG CCA  < 960
F   N   Q   S   M   T   E   N   H   L   E   A   G   M   V   I   T   D   E   P
GGA ATT TAT ATT CCT GAA TTT GGT GGA GTC AGA ATT GAA GAT GAC TTA TTA GTG ACA GAA  < 1020
G   I   Y   I   P   E   F   G   G   V   R   I   E   D   D   L   L   V   T   E
```

```
                                                                XhoI
AAT GGC TGT GAA GTC TTA ACA AAA GCC CCA AAA GAG CTT ATC GTT ATT CTC GAG CAC CAC  < 1080
N   G   C   E   V   L   T   K   A   P   K   E   L   I   V   I   L   E   H   H
CAC CAC CAC CAC TGA GAT CCG GCT GCT AAC                                          < 1110
H   H   H   H   *
```

Additional file 3. Nucleotide sequence of the *pepP* gene and the translated amino acid sequence from *Lactococcus lactis* ssp. *lactis* DSM 20481. Elements from the pET20b (+) are underlined. The restrictions sides (*NdeI* and *XhoI*) are in *italic*. The last amino acid of PepP is I. The amino acids L and E are originate from the *XhoI* restriction side, followed by the His_6-tag. The stop codon is signed with *.
