# Supplementary material for: Production, active staining and gas chromatography assay analysis of recombinant aminopeptidase P from Lactococcus lactis ssp. lactis DSM 20481
Source: AMB Express. 2012 Aug 1;2:39. doi: 10.1186/2191-0855-2-39 (PMC3418211; doi:10.1186/2191-0855-2-39)
Supplement: Additional file 4 — Sequences ofpepP/PepP. This file contains the nucleotide sequence of the pepP gene and the translated amino acid sequence from Lactobacillus plantarum NC8. (PDF 219 kb) [file 2191-0855-2-39-S4.pdf]

NdeI

GGA GAT ATA CAT ATG AGT CGA GTT GAA CGG TTA CAA AAT AAG TTT GAT CAG TTA AAA ATT < 60

M S R V E R L Q N K F D Q L K I

GAT GCG TTC TTA GTT TCA GAT GGG GCT AAC CTT CAA TAT TTA ACA GGA ATG GCT GAT ATG < 120

D A F L V S D G A N L Q Y L T G M A D M

GCC GGC GAT GGT TAT CTG CTA GTA CTG GCA CAG GAA GCC TAT CTG ATT ACG GAT GCC CGT < 180

A G D G Y L L V L A Q E A Y L I T D A R

TAT CAG ACG GCT TTT GCC GGT CAG TAT GAT GAC CAG CAC CTA GTC ATT ACA CGT GAT TAT < 240

Y Q T A F A G Q Y D D Q H L V I T R D Y

CTG GGG GCC GTT TGT GAT ATT ATT GCC AAA ACC GGT ACT GGG GTC ATG GGC TTC GAG GCC < 300

L G A V C D I I A K T G T G V M G F E A

GAG ATT CCA TAT ACA GCC TAC AGT TAC TTA GAT GAG AAC TTG GTC AGT GAT TTG GTT GCG < 360

E I P Y T A Y S Y L D E N L V S D L V A

TTA CCA GAC GTG GTC GAT GAG TTG CGC ATC ACC AAG AGT GTT GAT GAG ATT GAC CGG CTA < 420

L P D V V D E L R I T K S V D E I D R L

CGA GCG AGT GCA CGT TTG GCG GAT GCT GGT TTC GAA TAC GTA ACG AGT ATC GTG CGA CCA < 480

R A S A R L A D A G F E Y V T S I V R P

GGC ATG CGT GAA ATC GAC GTT AGC AAC CTG TTA GAC GCG TTT ATG CGG ACG CAC GGT GCA < 540

G M R E I D V S N L L D A F M R T H G A

AGT GGG CCG TCG TTC ACG ACA ATC GTA CTT GGT GGT GCA CGA GCG GCT TTA CCG CAT GGG < 600

S G P S F T T I V L G G A R A A L P H G

ACG GCT TCT AAA GCG CTC TTA ACT GCC GGG CAA CTT GTT ACT CTT GAT TTT GGT TAC TTT < 660

T A S K A L L T A G Q L V T L D F G Y F

TTG GAC GGG TAC ACG TCA GAT ATG ACA CGG ACG TTT GCT TTA GGT ACT CCG GAT GAC AAG < 720

L D G Y T S D M T R T F A L G T P D D K

TTG GTT ACG GCC TAC CAG GCG GTG CAG GCT GCT CAA CAG GCT GTG ATC GAT CAG GTC CAA < 780

L V T A Y Q A V Q A A Q Q A V I D Q V Q

GCA GGT GCA GCC ACT GCT CAA CTG GAT GCA GTT GGC CGC GAT TTG CTG ACA AAA GCG GGC < 840

A G A A T A Q L D A V G R D L L T K A G

TAT GGT GAC GCC TTC AAT CAT GGT ATG GGT CAC GGC ATT GGT CTT GCC ATT CAT GAA GGC < 900

Y G D A F N H G M G H G I G L A I H E G

CCG TTG ATC TCA AAA AAC ACG ACT GGC ACC TTA GTT GCA AAC AGT GTG ATT ACT GTT GAA < 960

P L I S K N T T G T L V A N S V I T V E

CCC GGG GTA TAT TTC CCA GAT TTA GGC GGA ATG CGG ATC GAA GAT GAT GTC TTA GTG ACT < 1020

P G V Y F P D L G G M R I E D D V L V T

XhoI

```
GCT GAG GGC CAT GAA CGA CTG ACG ACT GCA ACT CGT GAT TTA CTG ATT TTA CTC GAG CAC  < 1080
A   E   G   H   E   R   L   T   T   A   T   R   D   L   L   I   L   L   E   H

CAC CAC CAC CAC CAC TGA GAT CCG GCT GCT AAC                                      < 1113
H   H   H   H   H   *
```

Additional file 4. Nucleotide sequence of the *pepP* gene and the translated amino acid sequence from *Lactobacillus plantarum* NC8. Elements from the pET20b (+) are underlined. The restrictions sides (*NdeI* and *XhoI*) are in *italic*. The last amino acid of PepP is L. The amino acids L and E are originate from the *XhoI* restriction side, followed by the His_6-tag. The stop codon is signed with *.
